# Editorial Acknowledgments and Comments

**Published:** 1873-12

**Authors:** 


THROUGH the courtesy of the publishers, the *Atlanta Daily Herald* comes regularly to our reading-room files, for the use of subscribers and visitors. The youthful Editor and Manager of this very enterprising and "newsy" sheet, after a brief career as a newspaper correspondent, over the *nom de plume* of "King Hans," and while yet a boy in his "teens," with the energy and pluck so characteristic of him, purchased a tri-weekly newspaper at Rome, Ga., which he at once converted into a daily. In its editorial and business conduct he displayed precocious, but marked, ability; he won for his enterprise a handsome patronage, and when his soaring ambition conceived the idea of the *Atlanta Daily Herald*, with its mammoth issues, its special railway trains, and other features of startling *vim*, he turned over his more youthful enterprise into other hands; but it still survives, it yet comes daily to the doors of its patrons, and is still ably sustained.

It gives us pleasure to note the success of energy and pluck, especially in those just entering business-life. It is but just reward of enterprise and dilligence, and such example may well serve to stimulate the energy, the zeal, the industry of youthful members of our own profession.

---

THE PLANTATION.—Vol. 5, No. 1, for November, has been laid upon our table. We notice that this sterling agricultural monthly has passed into the hands of Col. C. R. Hanleiter, as editor, publisher and proprietor. We learn that after more than forty years of faithful toil at the case, in the press-room, and in general office management, he has espoused the *Plantation* as the *darling pet* of his old age. We also learn that he devotes his whole time to its regular production; that he edits, puts in type, revises, prints, publishes and mails it, with his own hands, and therefore, *knows that it is all right* before it leaves the office on its way to interested readers. We trust that those of our readers who are interested in agriculture, horticulture, or floriculture—and who is not?—will brighten up the declining years of the old veteran with their patronage and support. It is issued in monthly numbers of forty pages, at $1.50 per annum in advance.

